# Qualia and Phenomenal Consciousness Arise From the Information Structure of an Electromagnetic Field in the Brain

**DOI:** 10.3389/fnhum.2022.874241

**Published:** 2022-07-04

**Authors:** Lawrence M. Ward, Ramón Guevara

**Affiliations:** ^1^Department of Psychology and Djavad Mowafaghian Centre for Brain Health, University of British Columbia, Vancouver, BC, Canada; ^2^Department of Physics and Astronomy, University of Padua, Padua, Italy; ^3^Department of Developmental Psychology and Socialization, Padova Neuroscience Center, University of Padua, Padua, Italy

**Keywords:** phenomenal consciousness, thalamus, electromagnetic field, information structure, affordances, qualia

## Abstract

In this paper we address the following problems and provide realistic answers to them: (1) What could be the physical substrate for subjective, phenomenal, consciousness (P-consciousness)? Our answer: the electromagnetic (EM) field generated by the movement and changes of electrical charges in the brain. (2) Is this substrate generated in some particular part of the brains of conscious entities or does it comprise the entirety of the brain/body? Our answer: a part of the thalamus in mammals, and homologous parts of other brains generates the critical EM field. (3) From whence arise the qualia experienced in P-consciousness? Our answer, the relevant EM field is “structured” by emulating in the brain the information in EM fields arising from both external (the environment) and internal (the body) sources. (4) What differentiates the P-conscious EM field from other EM fields, e.g., the flux of photons scattered from object surfaces, the EM field of an electro-magnet, or the EM fields generated in the brain that do not enter P-consciousness, such as those generated in the retina or occipital cortex, or those generated in brain areas that guide behavior through visual information in persons exhibiting “blindsight”? Our answer: living systems express a boundary between themselves and the environment, requiring them to model (coarsely emulate) information from their environment in order to control through actions, to the extent possible, the vast sea of variety in which they are immersed. This model, expressed in an EM field, *is* P-consciousness. The model is the best possible representation of the moment-to-moment niche-relevant (action-relevant: affordance) information an organism can generate (a *Gestalt*). Information that is at a lower level than niche-relevant, such as the unanalyzed retinal vector-field, is not represented in P-consciousness because it is not niche-relevant. Living organisms have sensory and other systems that have evolved to supply such information, albeit in a coarse form.

## Introduction

The field of consciousness studies is too vast to be reviewed in a short paper. We will, accordingly, only mention a few important aspects of that literature for purposes of orientation to our proposal. First, we are sympathetic to the position of Searle (e.g., Searle, 2000) that phenomenal consciousness (P-consciousness or PC), our focus here, is a property of living, behaving, things. This is also consistent with the position of Gibson (1979/2014) that perception is a property of living, behaving organisms. We wish to avoid panpsychism for a number of reasons, not the least of which is its striking inconsistency with our everyday experience of rocks, BBQs, and even trees and grass (cf. Merker et al., 2021). Second, along with many others (e.g., Crick, 1994; Searle, 2000; Revonsuo, 2006; Fingelkurts et al., 2013), we assume that the fundamental substrate of consciousness involves nervous tissue, in humans the brain and the rest of the central nervous system. When the brain is dead PC is absent. And when the brain is severely injured, particularly the thalamus, PC is severely compromised and in some cases apparently absent as well (e.g., Jennett and Plum, 1972; Jennett et al., 2001; Ward, 2011). Therefore, we search for the physical substrate of PC in the workings of nervous tissue, especially in brains. Third, we agree with the majority of studies of PC in asserting that PC is a unitary, integrated, process that involves all of the senses as well as the emotions, bodily sensations, and semi-modal or amodal thoughts, although embodying different subsets of these elements from moment to moment (e.g., Edelman and Tononi, 2000; Seth and Baars, 2005).

## Physical Substrate for P-Consciousness

What could be the physical substrate for subjective, phenomenal, consciousness? Our answer: the electromagnetic (EM) field generated by the movement and temporal variation of electric charge in the brain. Since neural activity (and that of other aspects of the brain, e.g., slow flow of ionic currents in supporting fluids, activity across electrical synapses, etc.) consists of electrical charge movement (usually ions moving across cell membranes) and/or change in electric fields (as in ephaptic conduction, the coupling of neurons through electric fields), the answer must somehow be related to that activity. Moreover, synchronous neural firing is the normal mode of interaction of coupled neurons, since they are essentially relaxation oscillators whose nature is to be entrained by their inputs (Ward, 2002). Such synchronous (phase-locked, even at non-zero phase lags) firing has many consequences in the brain (e.g., Buzsaki and Draguhn, 2004). One of the most striking is the property that synchronously firing neurons reinforce each other’s effects whereas randomly asynchronously firing neurons cancel each other’s effects, both on each other and on downstream groups of neurons (cf. Fries, 2005, 2015). One consequence of synchronous firing is synchronous oscillations of electrical currents in the dendritic trees of the participating neurons, and synchronous oscillations of electrical currents in surrounding fluids and across neural membranes. It is important not to place too much emphasis on neural firing alone, even though spike potentials also generate EM fields. Axonal conduction of spike potentials is a neural communicative mechanism but may not be the most important aspect of neural activity related to consciousness. Several researchers have argued that electrical currents flowing in dendritic trees may be more related to consciousness and cognition than is neural firing, namely Mumford (1991; 1992), Pribram (1991); Nunez (2000), LaBerge and Kasevich (2007), and Fingelkurts et al. (2010, 2013). On the other hand, cortical spike potentials do encode information about sensory input, and even motor output, such as speech (e.g., Martin et al., 2018). But then, so do cortical local field potentials, including even imaginary speech (Proix et al., 2022). An important problem for any theory of PC based on EM fields is to describe which aspects of neural activity generate the EM field critical for PC. We will address this problem in several ways in what follows.

One important consequence of the charge flow/current change associated with synchronous (integrated) neural activity is that, according to Maxwell’s electromagnetic theory (e.g., Feynman et al., 1964; Hales, 2014), it creates an electromagnetic (EM) field that comprises all of the information expressed by that charge flow. As for electric fields within the brain, they are generated by electric charges according to Gauss’s law,


$$\nabla \cdot \vec{D}=4\,\pi \,\rho ,$$

where $\vec{D}$ is the electric field in matter (displacement field) and ρ is the charge density, and both are time-dependent functions. This implies that if the electric charges are changing with time inside the brain, the generated electric fields are also changing. The charges and currents include not only neural action potentials but also dendritic currents, axonal currents, and myriad other electrical and magnetic effects arising from neural and glial activity.

The sources of magnetic fields are, instead, electric currents, and they are also generated by temporal changes in electric fields:


$$\nabla \times \vec{H}=\frac{1}{c}\,\left(4\,\pi \,\vec{J}+\frac{\partial \vec{D}}{\partial t}\right),$$

where $\vec{H}$ is the magnetic field, $\vec{J}$ is the electric current and *c* is the speed of light. This implies that if currents are changing in time, magnetic fields are also changing in time. Finally, changes in time of electric and magnetic fields generate electromagnetic waves, as expressed in the previous equation and the equation:


$$\nabla \times \vec{E}=-\frac{1}{c}\,\left(\frac{\partial \vec{B}}{\partial t}\right),$$

where $\vec{E}$ and $\vec{B}$ are the electric and magnetic fields in vacuum, respectively. These equations, when applied to neural tissue, have a special form that is described in detail by Hales (2014). We cannot in this brief article describe in any more detail the physics of how EM fields are generated by neurons. Please see Hales (2014) for a detailed discussion of how neurons generate and sustain EM fields.

Maxwell’s equations have another important implication. They relate sources and fields in a deterministic way: once the sources are given, the fields can be uniquely calculated by solving the differential equations. So, if we know how charges are distributed and how they move in space (flows of charge), we know all about the corresponding electromagnetic field. *In other words, all the information contained in source configurations is also contained in the fields.* In this sense, the electromagnetic field “reflects” the sources, which are electric charges and their movement (currents). Or, to put it another way, the sources of the field create the *information structure* of the field.

Furthermore, since Maxwell’s equations are linear in the fields (the electric and magnetic fields enter linearly in the equations), their solutions obey the superposition principle. This has an important implication in the context of the current discussion, one that we think has not been sufficiently emphasized in the literature. The electromagnetic field generated by the activity of neurons is more integrated than are the sources themselves. Indeed, the integration of brain activity is mediated through neural synchronization that typically requires a physical interaction at synapses (except for ephaptic connections). But the electromagnetic fields generated in different parts of the brain, *providing they are close enough in space and time*, are automatically integrated, more or less independently of the anatomical connectivity of brain tissues generating them. For example, if two nearby neural sources, in points A and B, produce fields measured in C and D, due to superposition the fields in C and D are very similar, as they are the weighted sums of the fields that the individual sources (A and B) would generate independently. In other words, the fields are integrated, whereas the sources A and B may be integrated or not, depending on the connectivity between those sources. Additionally, the integration is established in very short time, instantaneously in comparison with physiological processes, as perturbations in electromagnetic fields are propagated at the speed of light. Thus, integration of information in an electromagnetic field is free of the temporal constraints of electro-chemical neural interactions, consistent with the phenomenal experience of a continuous flow of conscious experience (cf. James, 1890). Furthermore, phenomenal experience correlates with, and is possibly in causal relation to, information integration in neural circuits (e.g., Fries, 2005, 2015; Tononi et al., 2016). Integration of information between neural assemblies, however, can actually be achieved much faster by integration of their separate EM fields than by traditional synaptic mechanisms. These facts make the EM field a better candidate than the neuronal sources of the field as the physical substrate for consciousness, although of course still dependent on the activity of those neuronal sources for its generation (cf. also Fingelkurts et al., 2010 on this point).

If the synchronous neural activity taking place in the brain is closely associated with conscious awareness (Ward, 2011), then so, too, is the EM field created inevitably by that neural activity. Moreover, only the EM field integrates charge-flow activity into a unified spatio-temporal pattern that encompasses all of the information represented by the various aspects of the relevant neural activity. It is through their field effects that electrical events such as charges flowing across a membrane combine and interact in a smoothly integrated way.

Of course, this integration is time dependent and does not include all the brain, as the strength of an EM field from neural sources (typically, but not only, dipolar in nature) decreases rapidly with distance. At distances larger than a few centimeters, the activity of non-synchronized neuronal networks is indistinguishable from neural noise. An implication of this fact is that only highly synchronized neuronal assemblies contribute to an integrated EM field. In this sense, the EM field that we hypothesize to be a correlate of PC is the result of the activity of highly synchronized neural networks, similarly to other theories of consciousness (e.g., Dehaene, 2014). The most important difference of an EM based theory of PC from other existing theories is in the timing of integration. EM integration is, as we mentioned above, for all practical proposes instantaneous.

The fact that the EM field strength should be sufficient to contribute to PC is not the end of the story. It is the complexity of such fields that is associated with PC. For example, it is well known that during epileptic seizures the EM field is very large, as compared with the EM field in normal brain activity, but epileptic seizures are often accompanied by loss of consciousness, as is the case in generalized epilepsy. It has been shown that the complexity of functional networks of brain activity correlates with consciousness (e.g., Guevara Erra et al., 2016), and this fact should be reflected in the associated EM field. In other words, a necessary condition for an EM field to be relevant to PC is that it be both integrated and complex.

There is at least one problem remaining with our account: both electrical potentials and magnetic fields are volume conducted throughout the brain (Wolters and de Munck, 2007), in spite of the fact that these fields decrease rapidly in strength with distance from their source. The effects of dipolar sources add linearly, as mentioned above, and the mixtures can be detected by surface sensors such as EEG electrodes and MEG SQUIDs. Unmixing of these combined potentials is, in fact, an ongoing problem for noninvasive techniques in cognitive neuroscience (e.g., Wolters and de Munck, 2007; Delorme et al., 2012). Moreover, the various electrical and magnetic fields detected by EEG/MEG, and in particular those sourced to particular brain areas, are highly correlated with perceptual, cognitive, and motor behaviors. An appealing conclusion is that it is these global, integrated, EM fields that comprise PC. And indeed, most, if not all, researchers who propose EM field theories of PC argue that the relevant EM field is brain-wide, consistent with the linear mixing of local EM fields (e.g., Hales, 2014). In this paper, however, we discount brain-wide integration because most local processes are not represented in PC (see Section “Where in the brain? The thalamic dynamic core” for the argument). If our account is to be accepted, then, we must describe some process in the integration of the various EM fields that eliminates or cancels the fields generated by the local processes in favor of the resultant “final field” that represents all of the information our senses, memories, emotions, etc. are generating. There is clearly a tension between brain-wide integration of local coherent EM fields and formation of an integrated EM field in one central brain area of neural inputs from many local areas. In what follows we attempt to address this tension, although at this point we cannot offer a quantitative argument.

Thus, we propose, with several others (Pockett, 2000; John, 2001; McFadden, 2002, 2020; Fingelkurts et al., 2010, 2013; Hales, 2014), that an EM field generated by the brain (or even by some less complicated nervous systems) is directly involved in conscious awareness, indeed it *is* phenomenal consciousness. Proposals that some sort of EM field is conscious awareness have not received much attention previously. As mentioned, one serious problem has been that it is difficult to disentangle the parts of the brain’s EM field that reflect conscious awareness from those that reflect unconscious information processing. Some previous proposals implicate the entire EM field of the brain in PC (Pockett, 2000; Hales, 2014), whereas others argue that the EM field relevant to PC is generated in specific parts of the brain (this article; John, 2001) or in respect of a specific kind of brain activity (McFadden, 2002, 2020), and yet others propose a nested hierarchy of EM fields in the brain (Fingelkurts et al., 2010, 2013). We will address this problem directly in Sections “Where in the brain? The thalamic dynamic core” and “The Conscious EM Field.”

## Where in the Brain? The Thalamic Dynamic Core

Is the substrate of PC generated in some particular part of the brains/nervous systems of conscious entities or does it comprise the entirety of the brain/body? Our answer: a part of the thalamus in mammals, and homologous parts of other brains, generates the critical EM field.

Ward (2011) argued that the substrate for PC is located in the thalamus of the brain. The argument rested on four “pillars” of evidence. Here we only adumbrate Ward’s (2011) argument – please see that paper for detailed argument and more references. First, and perhaps most important, is the fact that PC is restricted to the *results* of cortical computations; the computations themselves do not enter PC. These results constitute a dynamic (ever-changing) core of integrated neural activity associated with PC (cf. Kinsbourne, 1988; Edelman and Tononi, 2000). Ward (2011) provided numerous examples of this fact, one of the most salient being that the extensive computations required to analyze retinal input into a variety of feature maps and then reconstitute these maps into a visual percept (e.g., Marr, 1980; Treisman, 1988) are never available to PC. As Gibson (1979/2014) described it, visual perception is “direct,” meaning that we see our visual environment without any awareness of the many intervening processes taking place. We now know much from a third person perspective about these processes (e.g., Coren et al., 2004) but do not experience any of them. The same applies to perceptions arising from all other sensory systems, including endogenous systems, memory retrieval, speech encoding and decoding, and even to thinking (see Ward, 2011 for discussion). As we will discuss later, these results are presented in PC as a niche-relevant view of the world combined with associated thoughts and emotions, which does not need to contain any of the myriad complicated physiological processes that give rise to them in order to adaptively guide behavior.

Second, the thalamus is deeply involved in the action of all common general anesthetics (e.g., Alkire and Miller, 2005), and is typically dysfunctional in patients with unresponsive wakefulness syndrome (UWS), in which a patient evidences sleep-wakefulness cycles but never responds to any external stimuli while “awake.” The case of Karen Ann Quinlan is a striking example of the latter. She persisted in UWS for 11 years after a drug-alcohol interaction caused a cardio-pulmonary arrest. Upon autopsy it was discovered that her cortex was fundamentally intact, but her thalamus had suffered significant damage from hypoxia. Other studies of similar incidents point to involvement of the thalamus in nearly all cases. In particular, the dorso-medial nucleus is especially sensitive to damage, with loss of 30% or more of its neurons always associated with UWS (Maxwell et al., 2004). Finally, tissue atrophy in the thalamus is strongly associated with the signs of awareness upon which clinical diagnoses depend, in contrast to atrophy in the basal ganglia, which is associated with clinical signs of wakefulness (Lutkenhoff et al., 2015). Moreover, nontraumatic brain injury (e.g., anoxia) causes more extensive atrophy in the thalamus, with accompanying UWS, than does traumatic brain injury (Lutkenhoff et al., 2015).

Third, the anatomy and physiology of the thalamic neurons, particularly that of the dorso-medial nucleus, are ideally suited for an integrative role. The excitatory neurons have extensive, branching dendritic trees populated by many different types of synapses. Except for the basic sensory nuclei (lateral geniculate, medial geniculate, etc.), they receive all of their input from the cortex, and about 90% of the traffic over cortico-thalamic loops is from cortex to thalamus, only 10% from thalamus to cortex. It seems that the cortex is downloading the results of its computations to the non-sensory thalamic nuclei (cf. Mumford, 1991). Notably, the dorso-medial nucleus receives inputs from nearly all areas of cortex and sub-cortex and is ideally suited to integrate all of this information into a charge flow that would result in a structured EM field comprising it all. Moreover, the dorso-medial nucleus is implicated in numerous neuropsychological disorders, particularly memory disorders (Ward, 2013).

Fourth, neural synchronization is a fairly well-established cortical neural correlate of PC (e.g., Cosmelli et al., 2004; Doesburg et al., 2009), and the thalamus is also a primary source and controller of synchronization, both in cortex and itself through the matrix neurons found in all higher-order thalamic nuclei (e.g., Barth and MacDonald, 1996; Jones, 2009). Synchronization of oscillations in several, now-canonical, frequency bands (theta, alpha, beta, gamma), generated by populations of neurons, has been associated with modulation of numerous cognitive and behavioral tasks in both humans and other animals (e.g., Varela et al., 2001; Ward, 2003; Buszaki, 2006; Womelsdorf et al., 2007; Palva, 2016). It has also been argued to mediate information transfer throughout the cortex (e.g., Fries, 2005, 2015; Buehlmann and Deco, 2010; Akam and Kullmann, 2014; Quax et al., 2017). Moreover, synchronization-mediated information transfer certainly involves the thalamus, or at least the pulvinar nucleus (e.g., Saalmann et al., 2012; Quax et al., 2017; Jaramillo et al., 2019). Thus, Ward (2011) argued that a thalamic dynamic core of synchronized neural activity, perhaps principally in the dorso-medial nucleus, constitutes the physical substrate of PC.

Merker (2012) discussed the problem of integrating the various sensory and non-sensory neural codes generated in the brain. According to the idea that the brain minimizes free energy by performing predictive coding based on a hierarchy of Bayesian probabilistic operations (Hinton and Sejnowski, 1983; George and Hawkins, 2009; Friston, 2010; Safron, 2020), the lingua franca of the brain is likely to be those probabilities. But we don’t experience probabilities. We experience the environment at a niche-relevant scale. Therefore, there must be someplace in the brain where all of the probabilities collapse into percepts, a kind of “winner-take-all” process regarding the various possible states of the world based on incoming sensory information and previous learning. This has been called the problem of “Bayesian blur” (Lu et al., 2016; Clark, 2018). Merker (2012) proposed that the pulvinar nucleus of the thalamus was likely to be such a place, where a “best estimate buffer” integrated and imaged the results of all of the probabilistic computations performed in the cortex. Merker’s work places the ideas of the Gestalt psychologists firmly into a modern framework. Merker (2012) also argued persuasively that only a few million neurons – maybe even 1 million – would suffice to generate the relevant human PC field. The pulvinar nucleus comprises several million neurons, as does the dorso-medial nucleus. Thus, either would suffice to support Merker’s best estimate buffer.

Another interesting approach to dealing with the problem of the Bayesian blur was the suggestion of Dehaene (2014) that the “collapse” of the Bayesian probability distributions could be likened to the probabilistic collapse, or reduction, of the deterministically evolving wave function in quantum mechanics (see also Safron, 2022). The wave function, comprised of a superposition of all of the possible state trajectories of a quantum system in phase space, is, in the Copenhagen interpretation, caused to “collapse” into a “real” state by the act of “observation.” This is similar to the superposition in the Bayesian brain of all of the possible brain states based on the current external and internal context, the Bayesian blur, and the subsequent collapse of these possibilities into an actuality, corresponding to a “real” percept, a thought, etc. This metaphor is suggestive, and is also similar to the idea of “objective reduction” (OR) of the wave function proposed by Penrose (1989), and elaborated by Penrose (1989) and by Hameroff and Penrose (1996) into a theory of quantum computation in the brain that is the basis for PC. This theory is beyond the scope of the present paper, but we note that the hot (in the quantum sense) environment in the brain is thought to cause quantum wave function reduction far faster than would permit the mechanism suggested by Hameroff and Penrose (e.g., Tegmark, 2000). Nonetheless, it is possible that quantum theory could be applicable to PC in some way. For example, the EM field is quantized, and so a quantum formulation of EM information integration could prove to be enlightening. Perhaps such a formulation would lead to a more explicit description of the computational role of EM fields in PC.

Jerath and Crawford (2014) assembled evidence from contralateral neglect syndrome and other sources that implicated the thalamus in PC of 3D space. They proposed, similarly to Ward (2011), that the thalamus integrates “processed information from corticothalamic feedback loops,” and also that the thalamus “reimages” visual and other sensory and non-sensory input in a dynamic virtual 3D space in the “mind.”

Rudrauf et al. (2017) proposed a mathematical theory of the spatial field of PC in which projective transformations and active inference (predictive coding) play an important role. In their theory, point of view is informed by projective transformations that integrate memory, expectation, and sensory input. Similar to Jerath and Crawford (2014) they postulated a virtual Cyclopean eye located behind the eyes in the center of the head without taking a position on the location or composition of the neural topology that supports it. Point of view is also implicated in the Gibsonian approach (1979/2014, Section “Qualia”) – the niche relevant point of view is that of affordances for action which would be from someplace in the body. Because the eyes in the head can “see” positions and movements of limbs and trunk (feedback from movements) the best visual point of view is from somewhere in the middle of the head.

The thalamic dynamic core and similar proposals just discussed make it possible to separate brain activity that directly gives rise to PC from other, supporting, brain activity that remains unconscious. These proposals separate processing that computes the *contents* of consciousness (cortical) from that which *displays* consciousness itself (thalamic). Thus, we propose that the thalamic dynamic core entails that the critically relevant EM field for conscious experience is the one generated by the synchronous neural activity in the thalamic dynamic core. Interfering with this EM field, e.g., as would a lesion in the intralaminar nuclei (Bogen, 1995a,b), disturbs basic phenomenal experience. Interfering with the EM field generated by the cortex, as does, for example, trans-cranial magnetic stimulation (TMS) or a lesion caused by a localized stroke, tumor, or accident, generally only affects the contents computed by the affected cortical area(s).

There are other, somewhat different, points of view on this question, however. For example, Fingelkurts et al. (2010, 2013) argued that the different parts of the brain each create their own EM fields (characterized by them as EEG fields), and that these participate in a cortex-wide hierarchy of interacting fields. In their view the highest-level phenomenal scene is composed of phenomenal objects that are in turn composed of phenomenal features, each of which arises from activity at its own level of the hierarchy (similar to the doctrine of specific nerve energies – see the discussion in Section “The Conscious EM Field”). So, in a sense, all of the levels participate in creating, or are integrated into, the final phenomenal scene we experience, similarly to other theories of global EM field integration. Although we see much to recommend this view of a phenomenal hierarchy, in our view this approach still doesn’t answer the question of why the neural *processes* involved in creating each of these phenomenal levels are not experienced, as they are surely represented in the neural activity giving rise to the various phenomenal aspects. Therefore, we prefer the view adumbrated earlier in this section for why phenomenal experience does not include the neural processes that give rise to it.

Finally, there are several sophisticated theories that locate the critical physical processes generating PC firmly in the neuronal activity of the cerebral cortex. Involvement of the thalamus in these theories is usually in a supporting role of promoting computation in local regions, or influencing inter-regional synchronization (e.g., Dehaene, 2014; Tononi et al., 2016; Safron, 2020). Perhaps the most sophisticated of these is that of Safron (2020), who combines the integrated information theory of Tononi et al. (2016) with the neuronal global workspace theory of Dehaene (2014) in the context of predictive coding based on the free energy principle and an active inference framework (e.g., Friston, 2010). Safron’s (2020) approach is much too complex even to adumbrate here. The main argument, however, is that “…integrated information only entails consciousness for systems with perspectival reference frames capable of generating models with spatial, temporal, and causal coherence for self and world.” Here “consciousness” is meant to be PC. Access consciousness is provided by the integration with global workspace theory, in which various local processes interact in the service of a given conscious episode. Our approach is sympathetic to Safron’s (2020), especially in the reference to predictive coding and the perspectival reference frame. In Safron’s approach the contents of PC comprise an egocentrically organized visuospatial field, computed by predictive processing hierarchies, particularly in posterio-medial and inferior-lateral parietal cortices. This visuospatial field, embodied in neural activity, is certainly generating an EM field and this could be the EM field for PC. As we argued earlier, however, cortical processing is more likely to consist of probability distributions over possible states of the world. We would argue that these cortical computations would collapse in a winner-take-all thalamic EM field comprising the brain’s best guess as to the state of the world. This then, in our view, would be where the computed visuospatial field would be displayed. Safron argues, however, that EM fields in the thalamus, whereas they could be helpful in establishing synchronization manifolds and interregional communication, would not be sufficient on their own as a physical/computational substrate of consciousness. Thus, we are in disagreement here to the extent that, although we acknowledge the importance of cortical computations in creating the visuospatial field, we argue that this field as computed in the cortex is not the critical EM substrate for PC. Our main disagreement really rests on the fact that Safron’s approach does not explain why many, or even most, of the processes involved in computing the integrated information that is said to directly generate PC do not appear in PC. We see Safron’s approach as the most sophisticated treatment to date of the complex cortical computations that create the information content of the conscious field.

## Qualia

From whence arise the qualia experienced in P-consciousness? Chalmers (1996) argued that explaining experience (phenomenal consciousness) is the “hard” problem (as opposed to the “easy” ones of explaining cognitive mechanisms), and part of that is the classical philosophical problem of qualia. Why is red red? Why is the experience of sound different from that of light and both different from the smell of roses? How is it that neurons that are virtually identical in structure and function can create such a wide range of different qualia? Why is activity of some neurons in the auditory cortex associated with the experience of the sound of a symphony, whereas activity of the same types of neurons in visual cortex is associated with the visual experience of a painting? How is it that auditory thalamus can support *visual* behavior when retinal projections are directed there neonatally (von Melchner et al., 2000), or that parts of visual cortex in the early blind can support *tactile* processing of Braille (e.g., Sadato et al., 1996), or that parts of the auditory cortex support processing of *visual* stimuli in the early deaf (e.g., Finney et al., 2001)?

In our proposal, *sensory* qualia arise from the fact that the *dynamic EM field created by charge flow in the thalamus comprises the information structure associated with the environmental input to sensory systems.* Again, as described by Maxwell’s equations for electromagnetism, any movements of charge or changes in electric or magnetic fields in the physical world generate EM fields unique to, indeed structured by, those changes. In other words, such EM fields embody the information content of the moving charges or changing fields (cf. Wheeler, 1990; Grimes and Grimes, 2004). So, in vision, the flux of scattered and direct photons from any particular visual field that impact the retinae of the eyes, the so-called “isovist” (Benedikt, 1979, Figure 1), constitutes a distinct dynamic EM field (albeit only part of the EM field available at that place). In this case, several thalamic nuclei actually emulate that EM field with a thalamic EM field generated by charge flow computed by various visual circuits, mainly cortical, from photons incident on the retinae. Similarly, a structured dynamic EM field is generated in the brain when the sound waves registered by the mechanical actions of the peripheral auditory system stimulate the spiral ganglion and are then processed by the subsequent stages of the auditory system. This EM field comprises the information structure in the sound waves emitted by environmental vibrations characteristic of their sources, including the frequency spectrum and its changes over time. And so on for the other senses, including sensations of movement, pain, pleasure, effort, and thinking (although some of these may be amodal qualia – see later).

**FIGURE 1:**
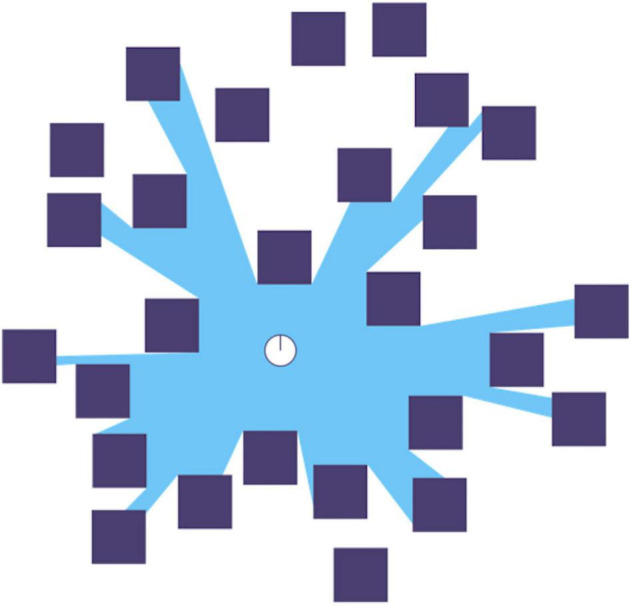
A representation of a 2D isovist in blue relative to the white dot in the center: “A single isovist is the volume of space visible from a given point in space, together with a specification of the location of that point.” (Wikipedia). Note how superposition prevents some elements of the scene (some black squares and parts of others) from appearing in the isovist. Diagram from Wikipedia.

More precisely, and consistent with over 200 years of scientific study of sensation and perception (e.g., Gibson, 1979/2014; Coren et al., 2004), the *information structure* of the environment is (approximately) recreated by the brain in the thalamic nuclei (cf. Pribram, 1986; Wheeler, 1990; Chalmers, 1996; John, 2002). The “content of experience” *is* the information structure of the EM field being recreated, or actually emulated in the case of vision, in the thalamic nuclei. Subjectivity arises from the emulating process in a living brain.

What do we mean by “information structure”? Let us focus on vision. When the eyes are open, the spatial distribution of photons from the isovist (Figure 1) at any moment comprises a vector field incident on the retina. This is a coarse graining of the photon field scattered from the environment, and also collapsed from three dimensions (3D) to two dimensions (2D). Let us ignore the problem of reconstructing the 3D visual field and focus on the 2D vector field on the retina. We will also ignore time for now. Instead, we assume one small time interval over which the retinal vector-field is integrated/summed. The visual system first analyses and then synthesizes this retinal vector-field, preserving the topology of the retina and thus of the visual field. This analysis also preserves the distribution of wavelengths/frequencies (although only the ratio of long to short wavelengths is used at the highest level) via cone type absorption spectra, and overall density of photons (intensity) at each coarse-grained point. Molecules in rods and cones absorb photons, thus collapsing the integrated fields of those photons and using the energy therein to isomerize pigments and begin biochemical processes that result in generator potentials that stimulate various neurons in the retina, which in turn stimulate neurons further up the visual system and so on. As pointed out by Anne Treisman (e.g., Treisman, 1988) the visual system constructs many retinal-topology-preserving maps containing spatial information about various features, such as color, shape, movement, etc., but then must integrate these maps (in her theory by attending to a spatial location) into a percept as seen from a particular point of view.

The isovist (Figure 1) is defined relative to the positions of the eyes in the body – there are two of them for a typical person – one for each eye – the difference of the two isovists, each collapsed to 2D on the retinae and each from a slightly different point of view, contains information that allows inference of the 3D isovist of a centrally placed eye (Cyclopean) from the separate 2D isovists. This must cohere with Rudrauf et al.’s (2017) theory and it does: point of view is a property of the isovist. The information structure of the EM field incident on the retina is comprised of the totality of photons, and their attendant properties, scattered by the elements of the environment within the isovist. We argue that a coarse-grained representation, or *emulation*, of this information structure is recreated by the visual system to produce the image we “see,” and that this is done in the thalamus (cf., Jerath and Crawford, 2014), likely in the dorso-medial nucleus (Ward, 2011). This approach is consistent with that of Merker (2012), who argues that this occurs in a “best estimate buffer,” although he locates it in the pulvinar nucleus of the thalamus.

In this proposal, the different experiential aspects of *sensory* qualia arise from the fact that the information structures produced in this way are unique to the particular aspect of the environment whose information structure is being emulated, for example, light (including wavelength, intensity, etc.), sound (frequency spectrum, temporal modulation, intensity), molecular vibration of taste and smell molecules, damage to biological structures (pain), and so forth. It all ends up as structured charge flow in the thalamus. Many aspects of higher-level computations, of course, do not have any corresponding environmental informational structure – they are unique to the computations performed in higher human cortex – and such computations will have unique, non-sensory, qualia. Some will be “amodal” (that is, having no sensory modality such as sight, hearing, etc.) and others will “feel” a certain way (perhaps because they would be associated with visual or auditory images), because the EM field created by the results of such computations sent to the relevant thalamic nuclei will “be” unique qualia. Emotional qualia will be blends of “visceral” qualia and “cognitive” qualia (cf. Damasio, 1999). The feeling of acting, of moving limbs and making effort, too, will be blends of various other qualia, both sensory and non-sensory. All of these EM fields must share the special character that differentiates conscious fields from non-conscious ones. This notion is consistent with Edelman and Tononi’s (2000) description of a very large-dimensional “qualia space” in which qualia are represented by vectors. In this view, some of the subspaces of this space would be sensory or emotional, and some would be more abstract, having the “feel” of cognition. Some could even be “imaginary,” that is, associated with things that aren’t real, like the feeling of oneself flying over the ocean without being in an airplane.

## The Conscious Electromagnetic Field

What differentiates the P-conscious EM field from other EM fields, e.g., the flux of photons scattered from object surfaces, the EM field of an electromagnet, the EM fields generated in the brain that do not enter P-consciousness, such as those generated in the retina or occipital cortex, or those generated in brain areas that guide behavior through visual information in persons exhibiting “blindsight”? Our answer: living systems express a boundary between themselves and the environment, requiring them to model (coarsely emulate) information from their environment in order to control, through actions, to the extent possible, the vast sea of variety in which they are immersed (cf. Ashby, 1958; Dennett, 1991). This model, expressed in an EM field, *is* P-consciousness. The model is the best possible representation of the moment-to-moment niche-relevant (action-relevant) information an organism can generate, a *Gestalt*, or in Merker’s terms a “best-estimate buffer.” Information that is at a lower level than niche-relevant, such as the unanalyzed retinal vector-field, is not represented in P-consciousness because it is not niche-relevant. Living organisms have sensory and other systems that have evolved to supply such information, albeit in a coarse form relative to the information actually comprising their environment (e.g., de Vries and Ward, 2016; Feinberg and Mallatt, 2016; Morsella et al., 2016; de Haan et al., 2021).

The group of synchronously active thalamic neurons that constitutes the thalamic dynamic core produces, through its enormously complicated, synchronously oscillating electric charges, an EM field that must have some special characteristic possessed neither by the other, weaker and usually mutually incoherent fields produced by local circuits not joining in the dynamic core, nor by the myriad cortical circuits, also firing synchronously with the thalamic dynamic core, that are computing the contents being represented there. As mentioned earlier, it is difficult to discern what this special character could be. One possibility is that the field would need to be of a certain “strength,” although this would deny awareness to smaller animals, a position that is somewhat implausible given the evident similarities in brain structure among, e.g., mammals, and the complicated behavior of some non-mammals such as birds. Another possibility is that the conscious field would have some particular informational character, again, as mentioned earlier, perhaps related to the complexity and differentiation/integration properties emphasized by Sporns et al. (2000) and Tononi et al. (2016). Finally, an EM field arising from the synchronized behavior of millions of neurons would be unitary and reinforcing, whereas those arising from the isolated (although locally synchronous) activity of nuclei not integrated into the dynamic core would tend to cancel out, or at best inform the “fringe” of consciousness identified by James (1890).

Therefore, a question arises: why postulate an EM field rather than just complicated neural activity as the essential physical substrate of PC as has been done by many other researchers? Is dense neural activity alone not sufficient? If it were, then we must ask what aspect(s) of this neural activity creates and differentiate(s) the various qualia, especially sensory qualia?

Some Greek philosophers, such as Democritus (460—370 B.C.E.; cf. Russell, 1945), thought the answer was “eidola,” copies shed by perceptual objects that were carried up little pipes to a homunculus in the head that experienced them. In a way this answer was prescient of the view of Gibson (1979/2014), in that some aspect of the environment itself was thought to be entering the perceiver. Descartes (1664) proposed a mechanistic view in which “motions” in the world were translated by the senses into “motions” in the body machine that were related to the motions in the world. These motions were then experienced in the mind via the pineal gland. An even more sophisticated viewpoint, one that prevailed far into the modern era, was the “doctrine of specific nerve energies,” in which different sensory nerves conducted to consciousness their own state, not, at least not directly, the state of the external world (Müller, 1835). Here, each type of sensory nerve had its own “specific nerve energy” that equated to the sensation it produced in the observer. Thus, activity in visual nerves would be “seen,” activity in auditory nerves would be “heard,” activity in gustatory nerves would be “tasted,” and so forth. The fact that visual nerves are connected to light sensors, etc., was the connection to the external world. This latter appears to be the view of some researchers still, e.g., Haikonen (2020). In Haikonen’s view, qualia equate to “self-explanatory information,” which arises from basic “sensory percepts” or their mental analogs. In Haikonen’s robot these “meanings” are analog electrical signals from sensors and effectors that are associatively linked to produce pattern recognition, memory, etc. In humans, we gather, they would be the (unspecified, analog?) neural activity produced locally in visual, auditory, etc. systems. Fingelkurts et al. (2010) also might be said to subscribe to this view, although they emphasize the local EM fields as the substrates of the simple phenomenal qualia.

Our modern knowledge of neural information processing, however, has discounted the doctrine of specific nerve energies and its relatives as an explanation for sensory qualia. Indeed, Adrian (1926) showed that all motor and sensory nerves function in the same way, via electro-chemical energy, so that it would be impossible to tell from a recording of a stream of spike potentials whether they were occurring in a visual nerve, an auditory nerve, or, indeed in any part of the central nervous system that produces such potentials, unless one knew how the relevant information was encoded in the stream of spikes. He proposed that it was *where* in the brain a sensory nerve projected that made the difference in qualia experienced. But this cannot be the answer either, although still espoused by several researchers (e.g., Rolls and Treves, 2011). This is because, as mentioned in Section “Qualia,” the visual cortex can support inputs from either vision, auditory, or touch sensors, the auditory cortex can support those from either auditory or visual sensors, etc. In these cases, the qualia are those associated with the *input*, *not* those of the receiving area of cortex.

So what could be the alternative to the doctrine of specific nerve energies, or any of the other theories of qualia based on dense neural activity? There has to be some way sensory and other, derived, information is represented in the brain other than “which” neuron is firing, because sensory nerves operate similarly; pyramidal, stellate, etc. neurons are highly similar everywhere in the cortex, and thalamic neurons are similar in various nuclei as well. It must be “how” they are firing (or oscillating, or what is happening in charge flow within them and their dendrites, etc.) that represents the *information* sent from the receptors. This again recalls the idea of the Bayesian brain, where the neural activity represents probabilities of brain states, and thus states of the external (or internal) world (e.g., Hinton and Sejnowski, 1983; George and Hawkins, 2009; Friston, 2010). But how? Consider a color map in V2: it is a topological representation of which wavelength mix of photons is striking which part of the retina, and eventually originating from somewhere in 3D space. At the retina and in early visual areas this is somewhat ok – although opponent processes complicate matters. But when we get to Land territory (V4 and above), where all that matters is the ratio of long to short wavelengths to recreate the color distribution corresponding to photon wavelength across the entire visual scene, we are in trouble with any direct representation of wavelength. So how can the various qualia arise from various distributions of neural activity that are indistinguishable unless we know from outside what they represent? What is the fundamental neural correlate of qualia? We argue that it must be the way in which the neural activity represents, or emulates, the information structure of the relevant input, and that information structure is unique to the input itself.

As mentioned in Section “Physical substrate for P-consciousness,” Merker (2012) offered a solution to one aspect of this problem: that of how the brain manages to integrate the neural codes from very different sensory transducers and processors. He points to the idea that in the Bayesian brain it’s all about probabilities, a Bayesian blur. He argued that the cortex uses probabilities as its lingua franca but then, because we don’t “see” probabilities, must somewhere collapse these into conscious percepts, with a point of view, etc. Merker argued that the collapse occurs in the pulvinar nucleus, Ward (2011) has it in the dorso-medial nucleus, for still others it could be in somewhere in cortex (e.g., Safron, 2020).

## Conclusion

We have argued that in vision a complex EM field (photons) interacts with matter fields in the environment to generate a complex dynamic EM field that contains information (space-time distribution of frequencies and densities of photons) about the matter fields with which it has interacted. The eyes receive the photons from the isovist. These 3D dynamic EM fields are collapsed to 2D fields as they interact with the matter fields comprising the retinae, whilst preserving the topology of the isovist. The *information* contained in the complex retinal vector-field is analyzed and then synthesized by the neural structures of the visual system, and the synthesis is used to emulate the original complex external field within the brain (thalamus – DM nucleus?). A similar story can be told for the other sensory systems, although the environmental or somatosensory information is not generally presented to the receptors as EM fields, and also for cognition and emotion.

Our story, however, is obviously not complete (or detailed enough). According to Gibson (1979/2014) and to us, the *information* in the environment is what ultimately is responsible for the sensory qualia. So, an important question is: why do we see wavelength/frequency of photons as colors? Why do we hear sound frequency as pitch? Why does pain feel the way it does? Pleasure? Early Gibson emphasized direct perception of information from the environment, whereas later Gibson emphasized that the environmental information is used to compute affordances for action. So, then, why do we see, hear, etc., instead of just acting/behaving based on ambient information? Environmental information is rendered in niche-relevant form, which includes the effectors and egocentric position and motion of the actor, allocentric motion of parts of the environment, as well as environmental features relevant to goals, needs and security. If behavior actually begins in the brain *before* the triggering/relevant information is rendered in P-consciousness, as argued by, for example, Libet (1999) and Soon et al. (2008), *what then is the role of phenomenal consciousness?* It is becoming clearer what the role of the underlying brain activity giving rise to PC is, but why have the phenomenal experience at all when it occurs later? Some answers have been suggested, such as that PC is epiphenomenal, or that the P-conscious EM field (or dynamic core of neural activity) can affect processing in nearby or even distant parts of the brain, but most feel that there are serious problems with each of these answers. Thus, this question, part of Chalmers’ (1996) hard problem, remains unsolved.

## Data Availability Statement

The original contributions presented in this study are included in the article/supplementary material, further inquiries can be directed to the corresponding author.

## Author Contributions

LMW and RG wrote and edited the various drafts of the manuscript. Both authors contributed to the article and approved the submitted version.

## Conflict of Interest

The authors declare that the research was conducted in the absence of any commercial or financial relationships that could be construed as a potential conflict of interest.

## Publisher’s Note

All claims expressed in this article are solely those of the authors and do not necessarily represent those of their affiliated organizations, or those of the publisher, the editors and the reviewers. Any product that may be evaluated in this article, or claim that may be made by its manufacturer, is not guaranteed or endorsed by the publisher.
